# Differences in Gating Dynamics of BK Channels in Cellular and Mitochondrial Membranes from Human Glioblastoma Cells Unraveled by Short- and Long-Range Correlations Analysis

**DOI:** 10.3390/cells9102305

**Published:** 2020-10-15

**Authors:** Agata Wawrzkiewicz-Jałowiecka, Paulina Trybek, Przemysław Borys, Beata Dworakowska, Łukasz Machura, Piotr Bednarczyk

**Affiliations:** 1 Department of Physical Chemistry and Technology of Polymers, Silesian University of Technology, 44-100 Gliwice, Poland; Przemyslaw.Borys@polsl.pl; 2 Faculty of Science and Technology, University of Silesia in Katowice, 41-500 Chorzow, Poland; paulina.trybek@us.edu.pl; 3 Institute of Biology, Department of Physics and Biophysics, Warsaw University of Life Sciences—SGGW, 02-787 Warszawa, Poland; beata_dworakowska@sggw.pl (B.D.); piotr_bednarczyk@sggw.pl (P.B.); 4 Institute of Physics, Faculty of Science and Technology, University of Silesia in Katowice, 41-500 Chorzow, Poland; lukasz.machura@us.edu.pl

**Keywords:** glioblastoma, BK channels in plasma membrane, mitochondrial BK (mitoBK) channels, patch-clamp, hurst exponent, detrended fluctuation analysis (DFA), multifractal detrended fluctuation analysis (MFDFA), Ca^2+^-sensitivity

## Abstract

The large-conductance voltage- and Ca${}^{2+}$-activated $K^{+}$ channels (BK) are encoded in humans by the Kcnma1 gene. Nevertheless, BK channel isoforms in different locations can exhibit functional heterogeneity mainly due to the alternative splicing during the Kcnma1 gene transcription. Here, we would like to examine the existence of dynamic diversity of BK channels from the inner mitochondrial and cellular membrane from human glioblastoma (U-87 MG). Not only the standard characteristics of the spontaneous switching between the functional states of the channel is discussed, but we put a special emphasis on the presence and strength of correlations within the signal describing the single-channel activity. The considered short- and long-range memory effects are here analyzed as they can be interpreted in terms of the complexity of the switching mechanism between stable conformational states of the channel. We calculate the dependencies of mean dwell-times of (conducting/non-conducting) states on the duration of the previous state, Hurst exponents by the rescaled range R/S method and detrended fluctuation analysis (DFA), and use the multifractal extension of the DFA (MFDFA) for the series describing single-channel activity. The obtained results unraveled statistically significant diversity in gating machinery between the mitochondrial and cellular BK channels.

## 1. Introduction

The BK channels are ubiquitously expressed potassium channels which are characterized by a large conductance (ca. 300 pS) [1]. These channels are sensitive to membrane potential, concentration of Ca${}^{2+}$, Mg${}^{2+}$, heme and some other factors like temperature, or mechanical strain of the membrane [1,2,3,4,5,6,7,8]. These channels are encoded by the Kcnma1 gene wherever they are expressed. However, the BK exonic composition in different locations can vary due to the alternative splicing during the Kcnma1 gene transcription [9,10], or post-translational modifications [11,12,13]. This leads to a tissue-specific functional heterogeneity within the BK channels. Depending on the isoformal composition of the BK channels in a given location one can observe a versatility of such features like voltage/Ca${}^{2+}$ sensitivities, response to phosphorylation, etc. [9,10,11,12,13,14,15,16]. The phenotypic diversity of the BK channels can be also enhanced by the possibility of associating the channel protein with different types of regulatory β (1–4) and γ (1–4) subunits [14,17,18].

It has been shown that the mitochondrial variants of the BK channels (mitoBK) are expressed when the Kcnma1 gene undergoes splicing to the DEC isoform [19]. The DEC splice variant is recognized by the presence of an insertion of a 50-aa C-terminal sequence named after the three last amino acids, which prevents the expression of the BK channel at the plasma membrane. In so far as the localization of the BK-DEC splice variant of BK-type channels is confirmed in mitochondria, it is still not known whether this isoform imposes functional changes in channel’s activity compared to the other BK isoforms present in the cell membrane. Additionally, differences of biophysical properties between plasma membrane- and mitochondrial channels can result from various factors, including the presence of membrane-specific proteins or differences in the lipid composition of the inner mitochondrial membrane in comparison with the plasma membrane. The inner mitochondrial membrane is characterized by a high degree of unsaturation, the presence of cardiolipin, and the absence of cholesterol, whereas the plasma membrane is highly saturated, contains relatively large amounts of sphingomyelin and cholesterol [20]. These discrepancies between the mitochondrial and cellular membranes can also escalate the differences in gating between the BK channel isoforms located in the plasma membrane and their mitochondrial analogs [21,22,23,24,25]. Differences in the single-channel conductance can be observed, as in the case of the ROMK-like channels from the plasma membrane and their analogs in the inner mitochondrial membrane [26].

In this work, we analyze the differences in the single-channel gating dynamics between the BK channels from the inner mitochondrial and cellular membrane from human glioblastoma (astrocytoma, U–87 MG). The former reports [27,28], where the results of Western blot analysis, immuno-gold electron microscopy, high-resolution immunofluorescence assays, and/or polymerase chain reaction were summarized, determine the presence of BK channels in U–87 MG cell line and give strong evidence that only BK-β4 assemblies were present in the analyzed patches of the cellular and mitochondrial membrane. Moreover, the presence of BK channels was also confirmed by the use of paxilline and iberiotoxin which exerted a channel inhibiting effect in appropriate patch-clamp experiments. In turn, the application of BK channel opener NS1619 caused the increase of open state probability up to 0.95. Regulation of these channels by calcium were also confirmed [27].

Due to the fact that the BK channels are expressed in specific isoforms in glioma cells which differ from the other BK channel isoforms by the enhanced sensitivity to cytosolic Ca${}^{2+}$ concentration, they are called gBK channels [29]. The gBK channels in the plasma membrane play an important role in the physiology of glioma cells. They are involved in cell growth and extensive migration (also in glioblastoma stemlike cells), so they facilitate the invasiveness of glioblastoma [28,30,31,32,33]. Considering the putative physiological meaning of the mitoBK channels in gliomas, it was reported that they can be involved in the regulation of the respiratory chain [27]. Moreover, the involvement of mitoBK channels in the phenomenon of cytoprotection has been demonstrated in [34,35,36]. In consequence, activity of both groups of BK channels in gliomas is one of the component processes which renders this kind of cancer incurable so far.

From this perspective, the gBK channels seem to be a promising candidate as a drug target in novel therapies against glioblastoma, which is particularly important since even a combination of contemporary therapies (i.e., surgery, chemotherapy, radiotherapy, or even immunotherapy) turns out to be not sufficiently effective in glioblastoma treatment [37,38,39,40]. Development of highly specific modulators acting most effectively on the gBK channels within cell membranes could prevent shape and volume changes of glioma cells which would hamper triggering and motorizing their invasive migration in a crowded environment. Whereas targeting the mitochondrial BK channel variants may help to induce cell death by e.g., hampering its respiration. This kind of highly specific channel-targeted therapy is a challenge for molecular pharmacology. Nevertheless, to reach this milestone goal and avoid the side-effects [41,42], complex investigations should be carried out in a step-by-step manner, where subsequently the details of the whole molecular machinery of gating should be unraveled, and then possible active sites, as well as the highly specific ligands, should be indicated. We are convinced that the first task to realize that aim is to answer the question of whether there exist any differences in gating dynamics of BK channels from mitochondrial and cellular membranes, which we study in this work. The possible dynamical diversity can help to adapt the mitochondrial and cellular channels to the local conditions and ensure the maximal effectiveness of fulfilling their physiological role. In turn, the description of the changes between the patterns of conducting/non-conducting channel fluctuations can bring useful information which can be utilized in resolving the mechanism of switching between channel substates (relatively stable conformations).

The experimental data, which are analyzed here, are obtained by the patch-clamp method in a single channel mode at external conditions adjusted to impose possibly similar open probability levels of the channel for both cases—mitochondrial and cellular patches. Due to the significant differences between the Ca${}^{2+}$ sensitivity of the plasma membrane and mitochondrial BK channels indicated by us during a preliminary experimental set and confirmed by the studies from the literature [18,29,43,44,45], we applied different concentrations of Ca${}^{2+}$ in bath/pipette solutions to cover the open state probability variability range from below 10% to over 90% at the applied membrane potentials from −60 mV to 60 mV with a 20 mV step for both BK channel variants. To obtain a detailed picture of gating dynamics, first, we compare the recorded traces by the construction of voltage-activation curves. Then, we perform the correlation analysis of the time series of single-channel currents (all currents obtained experimentally, and the series of the currents corresponding to functionally open and closed channel separately) and the dwell-times of subsequent channel states (in three variants, as previously, i.e., all states, only conducting and only non-conducting ones). Despite this kind of analysis remains still quite unconventional in the field, we decided to apply it to the experimental data describing single-channel activity because of the large interpretative potential of the calculated characteristics. Following measures of the short- and long-range correlations are evaluated:

- the dependencies of mean dwell-times of (conducting/non-conducting) states on the duration of the previous state (here called “conditional mean dwell-times”),
- Hurst exponent by the R/S method [46],
- generalized Hurst exponent (scaling exponent) by the detrended fluctuation analysis (DFA) [47],
- spectral parameters obtained by MFDFA analysis [48].

The obtained results can be interpreted in terms of the complexity of the switching mechanism between stable conformational states of the channel of different pore-geometry and lifespan. Thus, our findings can indicate whether the splicing sequence corresponding to a particular location and/or the location-specific composition of the membrane can define the conformational machinery of the channel gating.

The Hurst R/S analysis describes a long-range memory effect in analyzed series [46]. This method was applied to the analysis of the single-channel activity in [49,50,51,52,53,54] where the authors analyzed series of dwell-times of subsequent channel states corresponding to experimental results, or the results of simulations of channel gating models which should reproduce this feature of the real system. It turns out that the analyzed series of channel states’ durations are long-range correlated. The short-lasting states tend to be followed by short ones, and long-lasting states - by long ones. This tendency is conserved over the range of time scales. The physiological meaning of this feature, as well as its origin, remain still not understood. A possible explanation of the long-range memory effect in channel dynamics was proposed in [54], where the authors postulate that the single-channel activity is highly biased by another independent process which occurs at a larger time scale in relation to the conformational diffusion of a channel protein, but still, it can deeply affect the diffusive space of the channel gate. A reasonable candidate for such a component process are fluctuations in membrane thickness near the channel’s location. Squeezing and relaxation of the membrane ought to detrimentally influence the mass density of the channel protein, and, thus, change the number of energetically equivalent substates within a given macrostate (conducting/non–conducting state).

The investigation of correlation properties of ion channel fluctuations by means of the Detrended Fluctuation Analysis (DFA) was previously described in [55] where the authors analyzed the series of dwell-times of subsequent channel states and in [56] where time series of single-channel currents were analyzed (the raw signal and, separately, series of those current values which correspond only to an open channel state, or only to a closed channel state). Both reports indicate persistent long-range correlations in ion channel gating and stronger correlation effect within the closed states than the conducting substrates of the channel [55,56]. The single-channel currents from the voltage-dependent $K^{+}$ channel in rat dorsal root ganglion neurons revealed the existence of memory effect by means of the DFA analysis [57].

The Multifractal version of the Detrended Fluctuation Analysis (MFDFA), where the basic DFA technique is extended over the range of statistical moments of the calculated variance in terms of the scaling function [48], was also used for the study of ion channel’s activity in [58,59]. The reported results addressing either dwell-time series of channel states [58] or single-channel currents [59] indicated that the channel gating is an orderly process exhibiting long-range correlation features of a multifractal form. The membrane potential strongly affects the obtained spectral parameters. The spectrum width corresponding to the experimental signal increases with the rising difference in electric potential on both sides of the membrane patch [59]. Such a result suggests that a higher complexity and entropy of the signal is reached both at strong membrane depolarization and hyperpolarization when compared to the recordings obtained at moderate membrane potentials. This, in turn, may be interpreted in terms of changes in attainable substates (stable conformations) at given experimental conditions, according to Boltzmann’s definition of entropy. Thus, one expects an increase in attainable channel’s conformations with the rise of the absolute value of membrane potential [59]. The system dynamics are substantially different in functionally open and closed states and the total signal recorded during experiments is affected to a larger extent by the nonconducting states than the conducting ones [59] (which is in agreement with the inferences from [55,56]).

What is worth noticing the aforementioned results and inferences pertaining to the analysis of the BK channels activity in the cell membrane. The Hurst, DFA, and MFDFA analyses were not conducted for the mitochondrial BK channels hitherto. Thus, the current investigations should supplement the literature in this aspect.

To gain possibly broad information about the channel dynamics from the correlation analyses performed in this study, we apply the Hurst R/S and DFA methods in investigations of both: series of channel currents and the corresponding dwell-times of subsequent channel states. These two approaches allow us to gain characteristics of channel gating dynamics which should be interpreted in a slightly different way. The current fluctuations should resemble the subtle changes of protein conformational substrates (at least between the conformations which are sufficiently remote from each other from the energetic and spatial point of view). Thus the correlation analysis of the single-channel currents should describe the memory effect in the process of switching between conformational states of different pore conduction (i.e., between those conformations that imply different pore geometry, and in consequence, different ability to transport $K^{+}$ ions). For persistent correlation, the high values of the current tend to be followed by high values in the consecutive time step and low values of the current tend to be followed by low values. In turn, during the R/S and DFA analyses of the dwell-time series the main focus of attention is on the character of the transitions between conducting and non-conducting states of different lifespan. The obtained correlation measures characterize the memory effect in switching within the spectrum of channel protein substrates occurring on a wide time scale range. In that case, the possible presence of long-range memory suggests that the short-lived substrates tend to be followed by the short-lived ones, and the long-lived substrates tend to be followed by the long-lived ones.

The former reports in the literature suggest that the memory effects may be observed both within the whole experimental series describing the channel’s fluctuations between the subsequent conducting and non-conducting states, and also independently within the functionally open and closed states separately [55,56,57,58,59]. Thus, in this work we analyze the series of channel currents and also the series of dwell-times of subsequent channel states in three variants: the total activity of the channel (which is a superposition of two alternate signals describing closed and open state dynamics), as well as the series where the original traces were divided into two signals, containing only closed or only open state fluctuations, respectively. In this way, we ought to get the information on whether the movements of functional domains within the channel protein that cause gating are correlated regardless of whether the channel is open or closed.

In the case of the MFDFA methodology, we only consider the raw experimental patch-clamp recordings due to the requirement for the high length of the analyzed series. Nevertheless, this kind of analysis has a valuable interpretive potential, since the changes in spectral width refer to the changes in attainable substrates of a channel (stable conformations) at given experimental conditions [59].

## 2. Materials and Methods

### 2.1. Cell Culture

The cell culture was carried out on a human glioblastoma cell line (astrocytoma U-87 MG) as given in [27,59]. Briefly, the cells were cultured in DMEM supplemented with 10% fetal bovine serum at 37 ${}^{\circ }$C in a humidified atmosphere with 5% CO${}_{2}$. The other substances used in the culture solution were: 2 mM L-glutamine, 100 U/mL penicillin, 100 mg/mL streptomycin. Every third-fourth day the cells were fed and reseeded.

### 2.2. Mitoplast Preparation

The experimental methods used for the preparation of fresh mitochondria and subsequent mitoplasts were based on differential centrifugation and hypotonic swelling, respectively, as described in ([27]). To obtain mitoplasts, the mitochondria were incubated in a hypotonic solution (5 mM HEPES, 100 μM CaCl${}_{2}$, pH 7.2) for approximately 1 min, and then a hypertonic solution (750 mM KCl, 30 mM HEPES, and 100 μM CaCl${}_{2}$, pH 7.2) was subsequently added to restore the isotonicity of the medium.

### 2.3. Electrophysiological Recordings

The experiments were carried out in mitoplast-attached single-channel inside-out mode for mitoBK channels and also in inside-out mode for the BK channels from the cellular membrane of glioblastoma. The currents were recorded using a pipette of borosilicate glass (Harvard, UK) with a resistance of 7–20 MΩ, which was pulled using the Narishige puller. The glass pipette was filled with an isotonic solution containing 150 mM KCl, 200 μM CaCl${}_{2}$, and 10 mM HEPES at pH 7.2 in case of the patch-clamp experiments on the mitoBK channels. In turn, bath and pipette solution contained 130 mM potassium glutamate, 5 mM KCl, 2 mM CaCl${}_{2}$, 2.03 mM EGTA, and 8 mM HEPES for the experiments on BK channels from the plasma membrane. In this way, the compositions of the pipette solutions ensured a similar level of Ca${}^{2+}$-activation of the investigated BK channels regardless of the differences in sensitivity to calcium ions between both examined channel’s isoforms. The current was recorded using a patch-clamp amplifier Axopatch 200B. The currents were low-pass filtered at a corner frequency of 1 kHz and sampled with Clampex software at a frequency of 4.00 kHz (at time intervals of 250.00 μs) in case of mitochondrial patches and 5.00 kHz (at time intervals of 200.00 μs) in case of cell membrane patches. Single-channel currents were recorded at fixed pipette potentials of −60, −40, −20, 20, 40, and 60 mV, at room temperature (22 ${}^{\circ }$C). The measurement error of single-channel currents was ΔI = 1$\times 10^{-6}$ pA (implied by the equipment). Each experimental time series comprised at least N = $2\times 10^{5}$ current values at the applied time resolution of the measurement (the maximal length of recording was N = $6\times 10^{6}$). At each value of membrane potential, we recorded time series of single BK channel currents using 3–7 independent patches for each cell membrane type. For each repeating patch-clamp experiment, a fresh patch of mitoplast or cell membrane was used.

### 2.4. Analysis of Experimental Data

#### 2.4.1. Construction of Dwell-Time Series

The construction of dwell-time series corresponding to the subsequent channel states from the original experimental data in a form of time series of single-channel current required choosing appropriate algorithm of event detection i.e., recognition and separation of conducting (functionally open) and non-conducting (functionally closed) state basing on the actual current value. Here we applied the procedure described in [60] to determine the threshold current value (TC) separating the conducting and non-conducting states. The main stages of the TC evaluation are as follows:

- plotting the probability density function (PDF) of ionic current approximated by the nonparametric kernel density estimate with Epanechnikov kernel with logarithmic scales on both the horizontal and vertical axes,
- finding the intervals, where the power-law scaling is satisfied,
- finding the point intersection of the power-law plots (between the unimodal densities representing functionally open and closed states of a channel). The current value corresponding to this point of intersection indicates the TC.

#### 2.4.2. Conditional Mean Dwell-Times of Conducting/Non-Conducting States

We investigated the conditional dwell-time distribution, i.e., the mean opening time duration, provided the previous closing time duration had some particular value (or mean closed time duration provided some duration of the preceding opening event). In this aim, we made use of 2D arrays (similar to histograms), where on the horizontal axis we put the preceding dwell-time duration, and on the vertical axis, we put the average dwell-time duration of a consecutive state. We assumed the time resolution of these arrays of 5 ms.

To understand how to construct such arrays, consider the conditional distribution of opening time duration. Having an opening event, we record the value of the preceding closed time duration. At a resolution of 5 ms, the corresponding array entry for the preceding closed time (the array index calculated as rounding down of t/5 ms) is incremented by the value of the opening duration. In the end, array entries are divided by the number of events accumulated in these entries, to obtain means. The uncertainty is estimated by a traditional uncertainty for the mean of the series aggregated from various realizations of electrophysiological recordings (to account for variability across patches).

#### 2.4.3. Hurst R/S Analysis

The Hurst exponent was calculated on the base of the original Hurst’s theory [46]. In brief, the procedure embraces the following stages: first, the original analyzed time series of *N* elements is divided into *n* non-overlapped segments of the length *s*. For each subseries, the mean value *E* and the standard deviation *S* are evaluated, and then, the corresponding mean adjusted series and the deviate cumulative series. For each modified subseries (i.e., deviate cumulative subseries) the range *R* is found as the difference between the maximal and minimal value within a given segment, and it is further rescaled by the standard deviation *S*. Then, the mean value of the <R/S> is calculated for the current resolution of the division of the original time series (*n* segments of the length *s*). Next, the whole procedure is repeated for different lengths *s* (here, the lengths of subseries are equal to the powers of 2 i.e., $s=2^{p}$, where *p* = 1, 2, 3, …). Finally, the Hurst exponent is estimated as the slope coefficient of a fitted straight line to a double-logarithmic dependence of form:

(1) $$\log{\left(\frac{R}{S}\right)}_{s}=H\log\left(s\right)+\log\left(c\right)$$

The values of the Hurst exponent can range from 0 to 1 and can serve as the classification criterion of a given time series in terms of its predictability. If the Hurst exponent takes the value of 0.5, it indicates the purely random behavior of the considered system. In that case, there are no correlations between the subsequent elements of the analyzed series. When 0<H<0.5 one can recognize antipersistent characteristics of a given series, which means that the adjacent elements of a given time series tend to switch between the values over and under the mean. If 0.5<H<1 that the analyzed time series exhibits trend-reinforcing behavior (its elements are long-term correlated) In this case one can anticipate that a positive increment between the adjacent elements will be followed by a positive one, and a negative increment will be followed by a negative one. The higher values of the Hurst exponent are obtained, the more evident is the trend-reinforcing behavior of the system.

#### 2.4.4. Detrended Fluctuation Analysis (DFA)

The procedure of the DFA analysis [47] (which extends the Hurst approach) determines a level of self-affinity (long-range correlations) of the data even in case of their nonstationarity (statistical characteristics change with time). Here inherent trends are removed at all time scales. First, the signal’s profile $y_{i}$ is estimated as the cumulative sum of the series $x_{i}$ with the subtracted average value 〈x〉

(2) $$y_{i}=\sum_{j=1}^{i}\left[x_{j}-\langle x\rangle \right]$$

Then, the $y_{i}$ is split into *n* equal non-overlapping segments of size *s*, which are equal to the powers of two. For each segment, one should find the local trend $y_{v,i}^{p}$. Here we did it by means of the linear least-squares fit (p=1). Then, for each *v*-th segment, the variance is calculated as:

(3) $$F^{2}\left(s,v\right)\equiv \frac{1}{s}\sum_{i=1}^{s}{\left(y_{v,i}^{p}-y_{v,i}\right)}^{2}.$$

and the detrended fluctuation function f(s) for a scale *s* is given as a square root of the average variance:

(4) $$f\left(s\right)=\sqrt{\frac{1}{n}\sum_{v=1}^{n}F^{2}\left(s,v\right)}$$

The detrended fluctuation function is calculated for different lengths of subseries *s*. Finally, the scaling exponent α is determined from the power-law scaling of the function f(s) with *s*. It is determined as the slope of the regression line of double-logarithmic dependence logf(s)∝αlogs.

The α exponent can be interpreted analogously to the Hurst exponent. Namely, if α = 0.5 one can recognize uncorrelated series. If the α lower than 0.5, the analyzed signal is antipersistent. The α values higher than 0.5 characterizes a persistent series. When the α≃1 the analyzed signal has 1/f noise characteristics. The values of α above 1 indicate the non-stationarity of the series.

The Hurst R/S and DFA analyses were applied to dwell-time series of subsequent channel states of the length $N_{T}=2^{12}$ (all states) or $N_{Sop/cl}=2^{11}$ (only open or closed states) and to series of single-channel currents of $N_{C}=2^{13}$ samples. The chosen length was dictated by the availability of data (e.g., the length of closed state durations was limited for the series obtained at membrane depolarization). If the original experimental series was sufficiently long, it was divided into non-overlapping subseries of desired length which were analyzed independently.

#### 2.4.5. Multifractal Detrended Fluctuation Analysis (MDFA)

The MFDFA technique can be considered as an extension of the basic DFA technique over the q−th statistical moments of the calculated variance in terms of the scaling function [48]

(5) $$G\left(q,s\right)=\left\{\begin{array}{cc} {\left(\frac{1}{2n_{s}}\sum_{v=1}^{2n_{s}}{\left[F^{2}\left(s,v\right)\right]}^{\frac{q}{2}}\right)}^{\frac{1}{q}}, & q\neq 0,\\ \exp\left(\frac{1}{4n_{s}}\sum_{v=1}^{2n_{s}}\ln\left[F^{2}\left(s,v\right)\right]\right), & q=0. \end{array}\right.$$

Consequently, the *q*-order generalized Hurst exponent H(q) can be calculated from the dependence $G\left(q,s\right)∼s^{H(q)}$ in a double-logarithmic scale. The mass exponent τ is given by the formula τ(q)=qH(q)−1. In the following step, the *q*-order Hölder exponent h(q) is calculated as a derivative of the mass exponent over the argument $h\left(q\right)=\frac{d\tau (q)}{dq}$. Finally, the *q*-order multifractal singularity spectrum D(h) (mf-spectrum) is estimated as a Legendre transform of the mass exponent

(6) D(h)=qh(q)−τ(q)=q[h(q)−H(q)]+1.

Here, we apply the focus-based formalism of the MFDFA developed by Mukli et al., see Ref. [61] for details. The MFDFA analysis was applied only to the experimental series of single-channel currents of the length $N_{MFDFA}=2^{15}$. Only in case of this kind of data could we ensure sufficiently long series to detect multiscale multifractal characteristics of the system. The dwell-time series are a composite series and consequently, they have a reduced length which precludes them to be analyzed by means of MFDFA methodology (in the whole range of membrane potentials for a typical duration of patch-clamp recording).

### 2.5. Statistical Analysis

Statistical analysis was performed using Statistica (v13.1PL) and the Pandas [62] statistical package dedicated to Python.

The significance level of $\alpha_{s}=0.05$ was selected for the performed statistics. The normality test calculated via Shapiro–Wilk formula does not allow us to confirm the hypothesis about the normal distribution for the majority of analyzed variables. Thus, in further analysis, the non-parametric analog of *t*-test for the independent samples in the form of Mann–Whitney U statistic was implemented.

## 3. Results and Discussion

### 3.1. Kinetic Features

**Figure 1:**
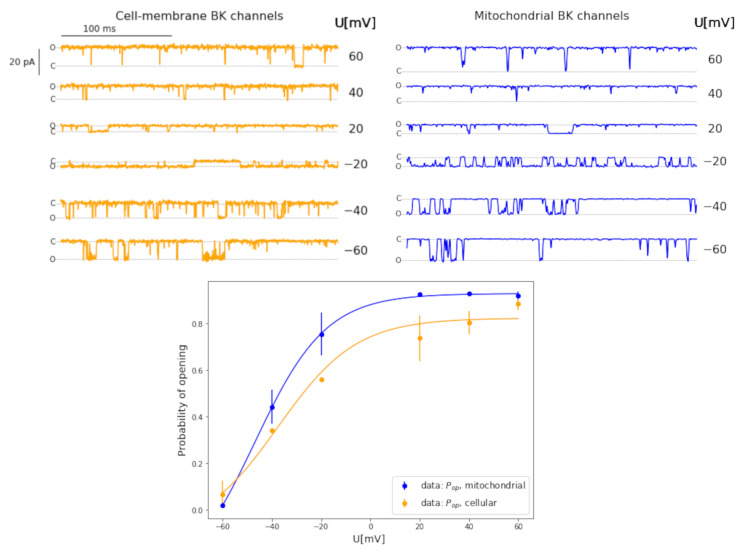
Representative experimental series of single-channel currents recorded on patches from cellular and mitochondrial membranes, and the corresponding voltage-activation curves. The empirical points representing the $p_{op}$ vs. $\left(U_{m}\right)$ dependence are fitted by a sigmoidal curve.

The samples of experimental patch-clamp traces and the corresponding activation curves ($p_{op}\left(U_{m}\right)$) are depicted in Figure 1.

Our results confirm that the electrophysiological features of different BK channel isoforms stay in a qualitative agreement with each other, both channels are regulated by membrane potential and Ca${}^{2+}$ concentration. Nevertheless, BK channels from the plasma membrane and their mitochondrial counterparts exhibit different sensitivity to calcium ions quantitatively. Namely, the mitoBK channels are significantly less sensitive to Ca${}^{2+}$ ions than the cellular ones. The application of the Ca${}^{2+}$ concentration of 200 μM in case of mitoBK and ca. 18 μM for their cellular analogs imposes a similar level of activation of the calcium sensors for both channel variants and, in consequence, allows to observe a possibly close variability of open state probability at the same range of membrane potential. For both analyzed groups of BK channels, the open state probability increased from below 0.1 to over 0.9 at the applied membrane potentials from −60 mV to 60 mV.

As one can see in Figure 1, the applied conditions allowed for a very good concurrence of open state probability at extreme analyzed voltages i.e., −60 mV and +60 mV for both channel variants. Some differences between the voltage-activation curves are visible when comparing the data from mitochondrial and cellular BK channels in glioblastoma at medium values of voltage. Namely, the BK channels from mitochondrial membranes are more sensitive to membrane depolarization than their analogs from the plasma membrane. We think that these discrepancies can stem from the different interactions between the voltage and/or calcium sensors and the channel’s pore-gate domain in case of the cell membrane and mitochondrial BK channel variants, or differences in lipid and protein composition of membranes which can further alter the channel gating. In fact, these differences in BK and mitoBK channel functioning were further investigated by means of several techniques of correlation analysis as a merit of this work. What is here also important, the applied measures of short- and long-term correlations do not directly depend on the average conductance of the channel. They refer to the structure of the signal and its complexity.

### 3.2. Conditional Mean Dwell-Times of Conducting/Non-Conducting States

**Figure 2:**
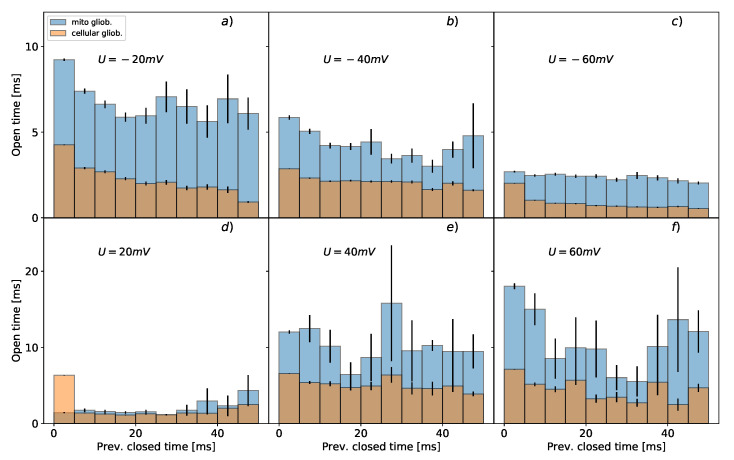
Mean conditional dwell-times of open states with error bars representing the corresponding standard deviations. The blue bars characterize the average values of conditional mean dwell-times for mitochondrial channels; the orange bars are assigned to the data describing BK channels from the plasma membrane. The upper panels (**a**–**c**) present the results obtained at membrane hyperpolarization; the lower graphs (**d**–**f**) correspond to channel characteristics at membrane depolarization.

**Figure 3:**
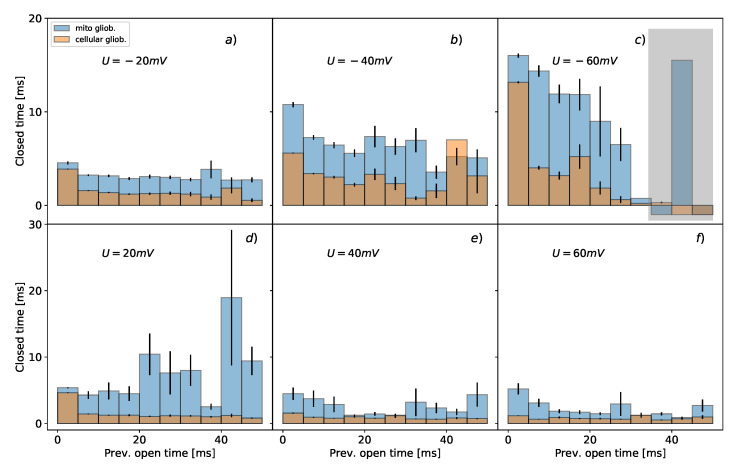
Mean conditional dwell-times of closed states presented with error bars representing the corresponding standard deviations. The blue bars characterize the conditional mean dwell-times for mitochondrial channels; the orange bars are assigned to the data describing BK channels from plasma membrane. The upper panels (**a**–**c**) present the results obtained at membrane hyperpolarization; the lower graphs (**d**–**f**) correspond to channel characteristics at membrane depolarization. The durations of closed states taking the value −1 (see gray rectangle) are assigned when a given previous length of open dwell-time was not recorded within a whole population of channel states.

The results of the conditional dwell-time duration distributions differ for the mitochondrial and cellular channels (Figure 2 and Figure 3). The open and closed time distributions differ on all positions of the preceding dwell-time for membrane potentials of −20 mV, −40 mV, and −60 mV. For positive membrane potentials, the differences are at more than 80% of considered positions. A general feature of the durations of mitochondrial channel states is that they can reach a longer lifespan than the ones describing the dynamics of channels within the cell membrane. The statistically significant differences between the distributions of dwell-time durations of mitochondrial and cellular BK channels’ states were identified for all membrane potentials except the open state at $U_{m}=20$ mV (*p* = 0.143) (Figure 3) and $U_{m}=60$ mV in the case of the mean closed dwell-time duration (*p* = 0.089) (Figure 2).

As a typical manifestation of voltage-sensitivity, in Figure 2 and Figure 3 there is a visible elongation of the open dwell-times with the increase of voltage, and a decrease of closed dwell-times when the voltage rises. One can also observe a lack of long-lasting open states at −60 mV (Figure 3).

As one can see in Figure 2 and Figure 3 at negative voltages long dwell-times of open states tend to be next to relatively short closed dwell-times, and vice versa. This kind of behavior is a manifestation of short-range correlations between the subsequent durations of consecutive channel states.

**Figure 4:**
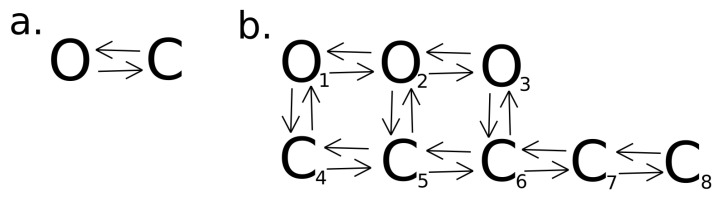
(**a**) A basic two-state Markovian model of channel kinetics. (**b**) A possible representation of BK channel kinetics by 3 open and 5 closed states.

To understand (without going into too many details) the meaning of the above analysis, let us consider some simple gating models. What is here well worth mentioning, the simplest possible description of the channel kinetics by a two-state Markovian model (see Figure 4a) is not able to describe any correlations between the dwell-times of channel states. The correlations can arise when channel kinetics is modeled by at least two substrates within the manifolds of open and closed channel states, and the open and closed states of the different average lifetime are connected to each other [63,64]. This can be manifested in a form of loops within the kinetic scheme which is shown in Figure 4b presenting a possibly simple representation of BK channel gating by 3 open and 5 closed states at fixed experimental conditions (that kind of modeling can be further extended to multiple state Markovian models to describe broad ranges of voltage and Ca${}^{2+}$ concentrations that affect gating).

In the general case, 3–4 open states and 5–6 closed states can model BK channel gating [65]. There are also several common multistate extensions of the Markov model [66,67], that allow for description of the gating kinetics in a broad range of voltages and concentration of calcium ions. In this paper, we do not evaluate the number of states or its connectivity based on the experimental results, but we would like to make the following remarks. Firstly, the kinetics of the channels from both mitochondrial and cellular membranes should be described by a relatively complex model with a large net of the open and closed substrates. Secondly, the recognized differences in conditional dwell-times of open and closed states are a direct consequence of the changes in the Markov diagram describing the considered channel isoforms. One can expect different sizes of the open and closed state manifolds or different rate constants for state transitions. In turn, the differences in Markov state diagrams are consequences of structural differences of the channel variants expressed in cell membranes and mitochondria that may affect interactions between pore-gate and the voltage/calcium sensors and gating dynamics (also different additional factors influencing conformational diffusion space of the BK channel protein may occur in mitochondrial and cellular membranes e.g., another lipid and protein partners which can interact with the BK channel).

### 3.3. Hurst R/S and DFA Analyses

Due to the fact that the DFA method can be considered as a generalization of the R/S methodology, we decide to provide the results obtained by both methods in one section. Admittedly, the DFA reveals more evidently the long-range memory effect than the R/S analysis. Still, the general inference, based on the Hurst and scaling exponents describing the single-channel activities from different patches is that both single-channel currents, as well as dwell-time series, unravel long-range memory effect for the BK channels’ activity regardless of the membrane type (mitochondrial or cellular), applied voltage, or whether we analyze the total signal or its components corresponding to functionally open or closed states. This makes that the existence of long-range correlations an inherent feature of gating dynamics.

It is difficult to indicate unequivocally the origins of the long-range memory in the channel’s activity. The discrete several-states Markovian models (3–, 4– and 11–state models) are not able to reproduce long-term persistence measured by the Hurst exponent [49,50]. Looking for an explanation of the causative factors inclining the Hurst memory effect, we support the previously formulated hypothesis [54], where the possible source of long-term correlation is related to the presence of a component side-process occurring at a larger time scale in relation to the conformational diffusion of a channel protein but still deeply influencing the diffusive space of the channel gate (like membrane fluctuations).

The recognized changes of the standard and generalized Hurst exponents with the increasing $U_{m}$ are relatively weak and non-monotonic. This may be surprising considering that at deep membrane hyperpolarization short openings tend to follow long-lasting closed states. At medium values of membrane potential both dwell-time durations equilibrate. Finally, as the voltage rises up to a high depolarization the dwell-times of channel states reach an extremum, where short closings tend to follow long-lasting open states. However, as stated above, it’s not the Markovian part of the channel dynamics that drives the long-term correlations.

From the reason that there is no clear tendency between the Hurst exponents and $U_{m}$ we grouped the sets of *H* (R/S) and α (DFA) exponents obtained for a given membrane-type at all the applied voltages and then compared medians of *H* and α representing mitochondrial and cellular patches in three variants: total signal, and the series of open and closed states separately.

#### 3.3.1. Analysis of Dwell-Time Series of Channel States

**Figure 5:**
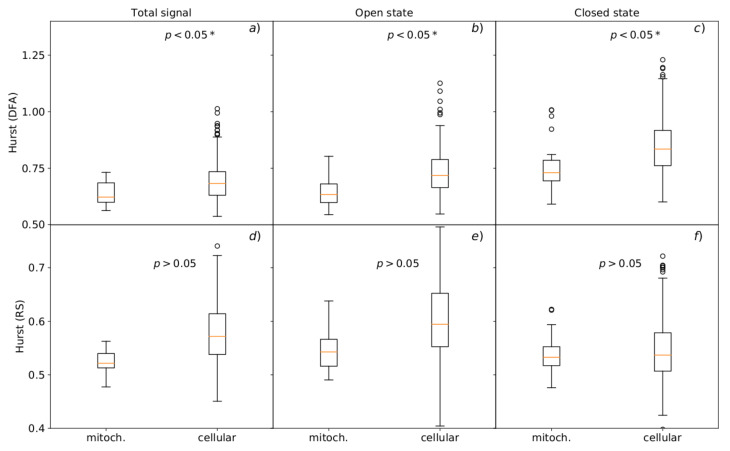
The Hurst exponents obtained by the standard R/S and the generalized DFA methods which describe dwell-time series of BK channel states for the channels located in mitochondrial and cellular membrane patches. The upper panels (**a**–**c**) present the results obtained at membrane hyperpolarization; the lower graphs (**d**–**f**) show the results corresponding to membrane depolarization. The boxes extend from the lower to upper quartile values. The line inside each box characterizes the median value and the whiskers represent the range of the data. Outlier points are those that exceed the ends of the whiskers.

The analysis of the dwell-time series of subsequent channel states by the Hurst R/S and DFA analyses allows for clear discrimination between the mitochondrial and cellular channels (Figure 5).

The memory effect is evidently lower for the channels from mitochondrial patches than for the cellular ones (Figure 5) regardless of whether total signal or openings and closings are separately analyzed. For the all-states variant (i.e., for the dwell-time series comprising both open and closed state durations) we obtained the Hurst exponents H=0.52,α=0.62 for mitoBK channels and H=0.57 and α=0.68 for cellular ones, which are given here as a median for the data obtained at all voltages. Analogous relation between the medians of *H* and α corresponding to mitochondrial and cellular channels holds for the cases when the closed and open states are analyzed separately. Namely, for the dwell-times of open states the H=0.54 α=0.63 for mitoBK channels and H=0.59 and α=0.72 for cellular ones, and for the dwell-times of closed states H=0.53 α=0.73 for mitoBK channels and H=0.53 and α=0.83 for the BK channels from plasma membrane.

Except for one case of the mean Hurst exponent values calculated by the R/S method for the closed state (*p* = 0.645 for Mann–Whitney U test, see Figure 5f) all the differences were statistically significant (p<0.05) when one compares the dynamics of channels from mitochondrial and plasma membranes.

Thus, one can observe that application of the R/S and DFA methods gave almost consistent results, but the recognized relation between the $H_{mito}$ vs. $H_{cell}$ is slightly better pronounced in the case of the DFA methodology (which possibly fits better to the structure of analyzed signals where the underlying statistics and dynamics can be non-stationary). The larger long-term memory effect between the dwell-times of subsequent channel states in the case of channels from cellular membrane indicate that in this case, the mechanism of switching between the states of different lifespan favors more evidently the pattern that the long-lasting sojourns in subsequent states should be followed by a long-lasting ones, and the short-lasting channel states should be followed by states of short durations. What is interesting this feature of the system is visible as a general rule occurring over a range of time scales (as dictated by the R/S or DFA methodology) except the short time scales, like in the case of the calculated conditional open and closed dwell-times (Figure 2 and Figure 3), where it disappears for both the mitoBK channels and their analogs form plasma membrane (this feature seems to be lost in the “Markovian” noise of the channel). The relation between the Hurst exponents, i.e., $H_{mito}$ < $H_{cell}$ may be interpreted according to the aforementioned framework of membrane thickness fluctuations [54]. Namely, a part of the fluctuations’ range may be insufficient to alter the diffusive space of the channel gate. In the case of the inner mitochondrial membrane, this fraction could be greater than in the case of the plasma membrane. In consequence, the fluctuations of the mitochondrial membrane seem to affect the mitoBK gating machinery to a smaller extent in comparison with the effects of plasma membrane fluctuations on the cellular variants of the BK channels.

What is also interesting, the deviations of the Hurst exponents describing BK channels from the cell membrane are significantly higher than those characterizing mitoBK channels (Figure 5). Thus, the memory effect has a significantly higher versatility for the plasma membrane channels—they are able both to present a strongly long-range correlated pattern of gating as well as antipersistent one. The presence of these outstanding subgroups of channel behaviors implicates that the population of cellular BK channel is not as uniform in terms of the characteristics of ionic signaling as the mitochondrial one.

#### 3.3.2. Analysis of Single Channel Currents

**Figure 6:**
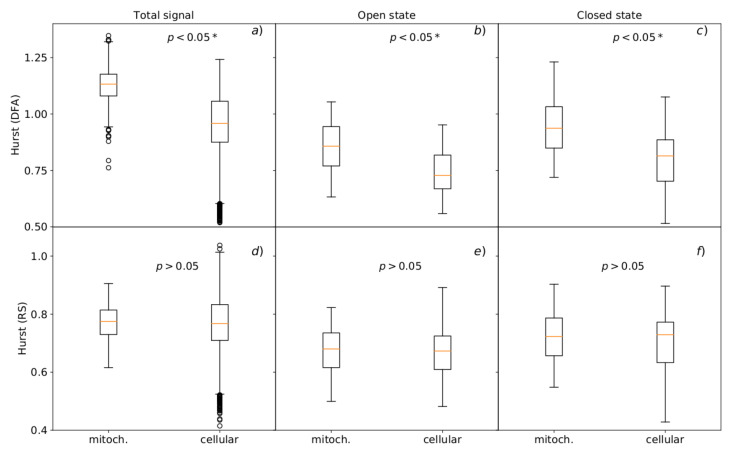
Hurst exponents obtained by the standard R/S and generalized DFA methods for the data describing single-channel currents of BK channels from patches of mitochondrial and cellular membranes. The upper panels (**a**–**c**) present the results obtained at membrane hyperpolarization; the lower graphs (**d**–**f**) show the data obtained at membrane depolarization. The boxes extend from the lower to upper quartile values. The line inside each box characterizes the median value and the whiskers represent the range of data. Outlier points are those which exceed the ends of the whiskers.

The persistent behavior of the system is even more evident (higher *H* and α exponents) in case of the analysis of single-channel current traces than the dwell-time series describing lifespan of subsequent channel states (analogously, like in the [55]), as shown in Figure 5 and Figure 6. Considering the results, the statistically significant differences of α exponents were obtained for all analyzed currents (*p* < 0.05) but only in the case of the DFA method (Figure 6a–c), in contrast to the R/S where there is a lack of sufficient grounds for rejecting the null hypothesis of no statistical differences between the compared variables: *p* = 0.440 in the case of the total signal, *p* = 0.239 for the currents recognized as channel openings and *p* = 0.123 for the fluctuations of recorded current during the closed state of a channel (Figure 6 d–f). These results can confirm the hypothesis that in relation to the R/S technique, DFA is a method that allows one to determine more subtle differences between the series characterized by a high degree of complexity.

For the currents corresponding to the closed channel’s states we obtained $H_{mito}=0.72$, $\alpha_{mito}=0.94$ and $H_{cell}=0.73$, $\alpha_{cell}=0.81$, and for the ones corresponding to the conducting ones: $H_{mito}=0.68$, $\alpha_{mito}=0.86$ and $H_{cell}=0.67$, $\alpha_{cell}=0.73$, where the exponents are given as medians from the data obtained at all membrane potentials.

As one can see the switching between the channel states of different pore conductance is realized in a more regular way (persistent) in the case of mitochondrial channels than for the ones located in the cell membrane. This effect can have its representation in the conditional dwell-time curves (Figure 2 and Figure 3) where in most cases the dwell-times of channel sojourns in the subsequent states reach higher values, so the mitochondrial channel tends to occupy conformations of similar conductance classified as one macrostate (conducting i.e., open or non-conducting i.e., closed) for a long time.

Similarly to the previous case when the dwell-times were analyzed, investigations of the long-range correlations within the single-channel currents also indicate that the total number of Hurst exponent outliers and the corresponding deviations are higher in the case of the cellular BK channels than the mitochondrial ones (Figure 6), which confirms the higher variability of gating behavior among cellular channels (a possibly more complex mixture of channel isoforms present in cell membrane than in the mitochondrial membrane; however the channels of outstanding characteristics are relatively rare—they do not affect the median considerably).

### 3.4. MFDFA Analysis of Experimental Single-Channel Recordings

**Figure 7:**
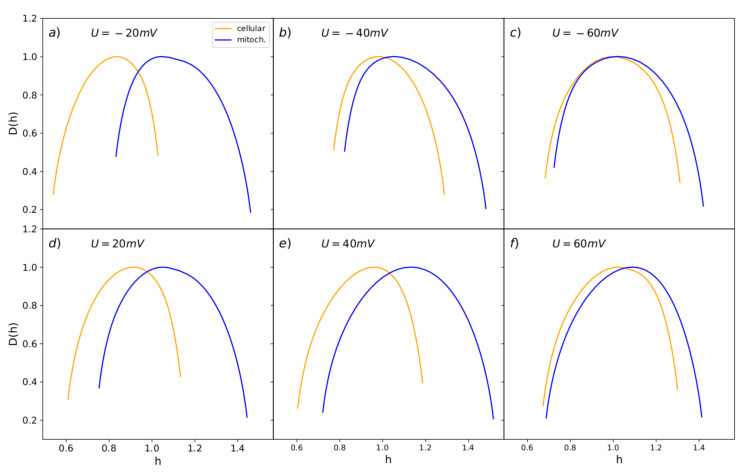
Average multifractal spectra obtained for BK channel from mitochondrial and cellular patches at different membrane potentials. The blue spectra characterize the mitoBK channel’s activity; the orange spectra are assigned to the BK channels from the cell membrane. The upper panels (**a**–**c**) present the results obtained at hyperpolarization; the lower graphs (**d**–**f**) correspond to the channels’ characteristics at membrane depolarization.

The final correlation analysis performed in this work was the MFDFA analysis of single channel currents. This methodology allowed us to indicate that the series of channel currents have multifractal characteristics and are nonrandom but caused by the orderly process exhibiting long-range correlation features regardless of the applied voltage or membrane type (cellular or mitochondrial). As one can see in Figure 7, the obtained multifractal spectra corresponding to BK channels from the inner mitochondrial membrane are well separated from the ones describing the activity of channels from plasma membranes. Only at strong membrane potential ($U_{m}=\pm 60$ mV) the gating dynamics of mitochondrial and cell-membrane BK channels seem to be less diversified.

**Figure 8:**
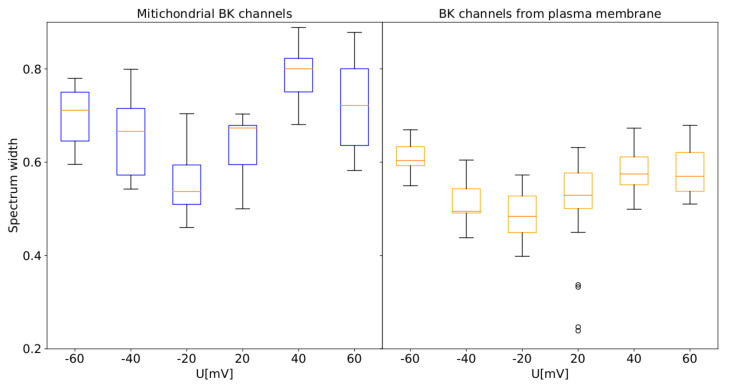
Box plots of the spectrum width obtained for the single-channel recordings for mitochondrial (**blue**) and cellular patches (**orange**) at different membrane potentials. The boxes extend from the lower to upper quartile values. The line inside the box characterizes the median value and the whiskers represent the range of data. Outlier points are those which exceed the ends of the whiskers.

Due to the fact that the spectral width (Δ) has the most influential interpretative potential than the other parameters of the spectra, we restricted the presentation of the obtained results to the Δ, as shown in Figure 8.

The obtained results show that the spectral width is evidently higher in the case of the BK channels from mitochondrial patches than the ones from the cellular membrane, which suggests a higher complexity and entropy of the activity of the mitoBK channel [68,69]. All the presented differences between the mf-spectrum width of cellular and mitochondrial series reached the statistical significance for the Mann–Whitney U test (*p* < 0.05).

According to Boltzmann’s definition of entropy (where the entropy is proportional to the natural logarithm from the number of real microstates of a considered system), these results ought to indicate a larger number of the attainable substates (stable conformations) in the case of the mitochondrial channel than its cellular analog. A hypothetical biological interpretation of this phenomenon is that the mitochondrial-membrane channels exhibit higher affordability to switch between the complex manifold of channel’s conformations, which can potentially improve their adjustment to an ever-changing environment.

The larger width of a spectrum at both strong membrane depolarization and hyperpolarization comparing with the ones obtained at moderate membrane potentials in both cases which can be a general feature of the BK channel dynamics regardless of its splice variant (cellular or mitochondrial), see Figure 8. From the definition of Boltzmann’s entropy, it seems that that recognized greater complexity of the signal at highly positive and negative potentials in comparison with the data obtained at membrane potentials close to zero should stem from a possible increase in the number of attainable channel substates with the absolute value of voltage. The qualitative agreement between characteristics of mitochondrial channels and the ones from the cell membrane suggests that this feature should stem from the interactions between the pore–gate domain and the voltage sensor (the structure of the membrane–spanning domains should be identical in both BK channel isoforms). In turn, the quantitative differences can originate mainly from different lipid surroundings within the plasma and mitochondrial membrane which can affect the BK channel conformational space.

### 3.5. Analysis of Randomized Series

To confirm whether the long-range memory effect recognized for the experimental data is its intrinsic feature, the investigated series were shuffled, and then the Hurst and scaling exponents as well as the MFDFA spectra were calculated for the randomized data sets. The randomization of analyzed series describing single-channel activity—both currents and dwell-times of channel states, caused the vanishing of the long-range memory effect measured by the Hurst exponent (H≈0.50±0.01 for all investigated cases). In turn, during the DFA analysis of shuffled series we encountered quite an interesting feature of the data. The randomization procedure applied to the analyzed series of single channel currents caused vanishing of the long-range correlations (α≈0.53±0.02 for all investigated patches). Nevertheless, a quite surprising result was obtained by shuffling the series of dwell-times of subsequent channel states. Namely, it does not fully erase the long-range correlations, but it allows us to significantly reduce them (all α values decreased by about 0.10). The possible explanation of this effect is that the distribution of dwell-times are not normal, and the dwell-times itself are conjunctive characteristics of the experimental signal. It turns out that the current work is not a sole report in which the long-range memory effect does not fully vanish after the shuffling procedure. In [49,50], as here, the Hurst exponent for the shuffled series was not exactly 0.50 (but about 0.57 ± 0.02). Still, most importantly, the application of statistical tests indicated that the Hurst exponent for shuffled data was significantly different from the mean value of the original experimental Hurst coefficient.

Shuffling operation of the series analyzed by the MFDFA method resulted in considerable changes of the spectral distributions and consequently affected significantly the spectral parameters (the spectra of shuffled data are about twice narrower). Reduced multifractality after mixing of the data gives clear evidence that the recognized fractal nature of the examined signals is its inherent feature and the system dynamics can be considered as an orderly process exhibiting long-range correlations.

## 4. Conclusions

In this work, the existence of dynamic diversity within the BK channels from the plasma membrane and the inner mitochondrial membrane in human glioblastoma cells is examined. Our results indicate that the mitoBK channels and their cell membrane analogs exhibit different sensitivity to calcium ions, which was observed or postulated by the other authors [18,29,43,44,45]. Probably the weakened sensitivity of mitochondrial BK channels stems from the structure-function relationship of the particular splice variants present in mitochondria or specific protein–protein and protein-lipid interactions within the inner mitochondrial membrane, which can deeply affect channel functioning and allows for the adaptation of channel transport efficacy to the local conditions; in particular relatively high Ca${}^{2+}$ concentration in mitochondria (which act as calcium reservoirs). It can be anticipated that the ligand sensors in both cases (mitochondrial and cellular BK channels) can exhibit other dynamics of calcium-binding. Probably, the Ca${}^{2+}$-sensors from the BK channels from plasma membrane can bind the calcium ions for a longer time than their mitochondrial analogs. This allows the BK channels from the plasma membrane to reach a low Ca${}^{2+}$ concentration (where the waiting time for the Ca${}^{2+}$ ions to approach the Ca${}^{2+}$ -free sensors is relatively large) a similar level of Ca${}^{2+}$ channel activation like the mitoBK channels at large Ca${}^{2+}$ concentration.

In the experimental part of our research, we attempted to adjust the external conditions to impose possibly close levels of voltage and calcium activation of the analyzed mitochondrial and cell-membrane channels to enable detection of possible differences in channel gating between these two isomeric BK channel variants.

We laid a special emphasis on the description of short- and long-range correlations within the experimental data describing single-channel activity. The obtained results allowed us to indicate both generic features of the gBK channels which are common for their mitochondrial and cellular variants (they are based on the qualitative agreement between the evaluated characteristics) and also point out the differences that allow us to discern both groups of channels (quantitative differences of evaluated measures between mito- and cellular channels). Among the inherent features of BK channel activity referring to the correlations in system dynamics are:

- short-range correlations between the lifespan of subsequent channel states confirmed by the analysis of the conditional dwell-time duration as shown in Figure 2 and Figure 3, which suggest the connectivity between the channel’s substates of different longevity (i.e., the number of routes between the substates within the manifold of functionally open or closed states) [63,64],
- long-range persistence indicated by Hurst exponents calculated by the standard R/S and DFA algorithms for single-channel currents (Figure 6) and the series of dwell- times of subsequent channel states (Figure 5),
- multifractal complex dynamics indicated by the MFDFA analysis (Figure 6), where the spectral width exhibits an analogous dependence on the membrane potential for both types of BK channels variants (broader spectrum at highly hyper- or depolarized membranes than at the $U_{m}$ close to zero) (Figure 8), suggesting similar changes of the conformational diffusive space with $U_{m}$.

These common characteristics can originate from the interactions and functioning of transmembrane segments of BK channels which should be almost identical for mitochondrial and cellular splice variants of BK channels in glioblastoma.

The performed analyses allowed also to indicate some of the details that distinguish the dynamics of mitochondrial and cell-membrane BK channels variants. The anticipated presence of some structural differences between the analyzed groups of BK channels resulted in e.g., the visible different Ca${}^{2+}$ sensitivity. For this reason, the experimental data used in this work were obtained at such conditions which ought to effectively attenuate these differences and impose a similar level of calcium activation of the channel. In consequence, we could observe the effects of interactions between the channel’s sensors within the channel isoforms from mitochondrial and cell membranes, and detect the possible subtle differences in the system’s dynamics.

The first evidence of such dynamical diversity was observed during the investigations of the conditional dwell-times of open and closed states of the channels. As mentioned in Results and Discussion Section this is a method for probing the Markov state diagram of the channel. Different states display different rate constants, so after estimating the mean time duration following a particular dwell-time, one may anticipate the connectivity between two states of the diagram. For example if one considers an open state duration that follows a closed state that lasts for 10 ms, one should investigate only the opening state, that is accessible from a closed state of liveliness 10 ms, but not from a state of liveliness of e.g., 30 ms. Obviously, if there are structural or functional differences in the channel, we expect the differences in the Markov state model that would describe such a channel. We have found differences in the conditional dwell-time distributions that indicate changes in the underlying Markov model, so the existence of the structural or functional differences between mitochondrial and cellular channels appears to be indirectly confirmed in that way. The questions that remain are: what is the origin of these differences? Is it only genetic splicing or folding conditions within the same genetic sequence? Possibly, the hypothetical slight changes of the dynamics of the Ca${}^{2+}$ binding, and further allosteric interactions between the sensors and pore-gate domains can also modulate gating dynamics (and the examined correlations). Another process that can influence gating kinetics can be connected with different compositions of cellular and mitochondrial membrane, and consequently other lipid-protein and protein–protein interactions [21,22,23,24,25]. An important side-effect of the conditional dwell-time analysis is that it should give good constraints to estimate the rate constants of the many-state BK channel models. This is, however, one of the problems that we would like to address in further research.

The analysis of long-range correlations indicates a larger memory effect within the series of dwell-times of subsequent channel states in the case of the BK channels from the cell membrane than the mitochondrial ones. This relation is observed irrespective of whether all channel states are considered or the openings and closings separately. Opposite relations are obtained when the series of channel currents are analyzed. The single-channel currents and the dwell-time series are however separate characteristics of the system, so such inversion of the relations is acceptable. What is worth mentioning it is that for all analyzed isoforms of the channel the long-range correlated characteristics of the non-conducting states of a channel determined the properties of the series describing the total trace of channel’s activity (higher *H* and α values at channel’s closings than openings in most cases), which is in good agreement with the former reports [55,56,59].

Summing up, the obtained results indicate that the splicing sequence or interactions with other membrane components corresponding to a particular location can define the conformational machinery of channel gating. This in turn allows us to adjust the channel functioning to a local environment and enable effective fulfilling of the physiological tasks of the channel in a given location. The recognized differences in gating dynamics between the channel variants can be represented as different connectivity, rate constants of transitions between the channels’ substates and/or even the number of attainable substates in appropriate kinetic schemes describing channel-gating (as in a popular Markov-type modeling), as postulated in this work. Considering the significance and potential utility of the obtained results this work shows that the methods of correlation analysis can serve as a useful tool to discern sets of experimental data describing the activity of channels that are structurally and functionally similar, as different groups of isoforms of the same channel type. Thus, the measures of short- and long-range correlations can be a basis for some classification algorithms in artificial intelligence approaches aimed to determine patterns within the experimental data describing ion-channel activity with or without the use of specific channel modulators. Their implementation in AI techniques (along with the measures of gating kinetics) can simplify the choice of the specific activating/inhibiting substances which modulate the channel functioning in the desired manner. The results obtained here can provide a theoretical background for further investigations on the potential modulators of the mitochondrial BK channels as well as their cellular analogs. As our outcomes suggest differences in gating machinery between the mitoBK and the BK channels from the plasma membrane in glioblastoma cells they allow us to infer that there should be a possibility of a highly specific modulation of only one group of them. Investigations of such kind of modulators could be one component in the development of new treatment strategies against gliomas which constitutes an important venture in future research.

Thinking beyond the glioblastoma treatment, one could pose also a general question about the possible utility of active substances which could affect the memory effects within the channel gating. Admittedly, the biological meaning of long-range correlations is still not fully understood. However, according to the definitions of the measures calculated in this work, this kind of therapeutics could play an important role in the regulation of biological processes whose characteristics reach a long-range in time. A reasonable candidate of diseases meeting this criterion are arrhythmia or epilepsy, for example. The potential meaning of BK channels in these diseases was summarized in the works [70,71] however the role of channel gating memory in their pathogenesis has never been discussed yet. One can anticipate that these diseases can be correlated with the abnormalities in long-range correlations in channel gating, which has to be confirmed in further studies.

## Abbreviations

The following abbreviations are used in this manuscript:

AI	Artificial Intelligence
α	the scaling exponent
DFA	Detrended Fluctuation Analysis
Δ	the spectral width
gBK	large-conductance voltage- and Ca${}^{2+}$-activated $K^{+}$ channel from the glioblastoma cells
H	the Hurst exponent
MFDFA	Multifractal Detrended Fluctuation Analysis
R/S	rescaled-range method of the Hurst exponent estimation
$U_{m}$	membrane potential

## References

[1] Marty A. (1981). Ca-dependent K channels with large unitary conductance in chromaffin cell membranes. Nature.

[2] Pallotta B.S., Magleby K.L., Barrett J.N. (1981). Single channel recordings of Ca^2+^-activated *K*^+^ currents in rat muscle cell culture. Nature.

[3] Squire L.G., Petersen O.H. (1987). Modulation of Ca^2+^-and voltage-activated *K*^+^ channels by internal Mg^2+^ in salivary acinar cells. Biochim. Biophys. Acta (BBA) Biomembr..

[4] Tang X.D., Xu R., Reynolds M.F., Garcia M.L., Heinemann S.H., Hoshi T. (2003). Haem can bind to and inhibit mammalian calcium-dependent Slo1 BK channels. Nature.

[5] Schubert R., Krien U., Gagov H. (2001). Protons inhibit the BKCa channel of rat small artery smooth muscle cells. J. Vasc. Res..

[6] Yang H., Zhang G., Cui J. (2015). BK channels: Multiple sensors, one activation gate. Front. Physiol..

[7] Wawrzkiewicz-Jałowiecka A., Dworakowska B., Grzywna Z.J. (2017). The temperature dependence of the BK channel activity–kinetics, thermodynamics, and long-range correlations. Biochim. Biophys. Acta (BBA) Biomembr..

[8] Allard B., Couble M.L., Magloire H., Bleicher F. (2000). Characterization and gene expression of high conductance calcium-activated potassium channels displaying mechanosensitivity in human odontoblasts. J. Biol. Chem..

[9] Tseng-Crank J., Foster C.D., Krause J.D., Mertz R., Godinot N., DiChiara T.J., Reinhart P.H. (1994). Cloning, expression, and distribution of functionally distinct Ca^2+^-activated *K*^+^ channel isoforms from human brain. Neuron.

[10] Fury M., Marx S.O., Marks A.R. (2002). Molecular BKology: The study of splicing and dicing. Sci. Stke.

[11] Schubert R., Nelson M.T. (2001). Protein kinases: Tuners of the BKCa channel in smooth muscle. Trends Pharmacol. Sci..

[12] Li M., Tanaka Y., Alioua A., Wu Y., Lu R., Kundu P., Sanchez-Pastor E., Marijic J., Stefani E., Toro L. (2010). Thromboxane A2 receptor and MaxiK-channel intimate interaction supports channel trans-inhibition independent of G-protein activation. Proc. Natl. Acad. Sci. USA.

[13] Shipston M.J. (2001). Alternative splicing of potassium channels: A dynamic switch of cellular excitability. Trends Cell Biol..

[14] Latorre R., Castillo K., Carrasquel-Ursulaez W., Sepulveda R.V., Gonzalez-Nilo F., Gonzalez C., Alvarez O. (2017). Molecular determinants of BK channel functional diversity and functioning. Physiol. Rev..

[15] Kyle B.D., Braun A.P. (2014). The regulation of BK channel activity by pre-and post-translational modifications. Front. Physiol..

[16] Poulsen A.N., Wulf H., Hay-Schmidt A., Jansen-Olesen I., Olesen J., Klaerke D.A. (2009). Differential expression of BK channel isoforms and *β*-subunits in rat neuro-vascular tissues. Biochim. Biophys. Acta (BBA) Biomembr..

[17] Contreras G.F., Neely A., Alvarez O., Gonzalez C., Latorre R. (2012). Modulation of BK channel voltage gating by different auxiliary *β* subunits. Proc. Natl. Acad. Sci. USA.

[18] Balderas E., Zhang J., Stefani E., Toro L. (2015). Mitochondrial BKCa channel. Front. Physiol..

[19] Singh H., Lu R., Bopassa J.C., Meredith A.L., Stefani E., Toro L. (2013). mitoBKCa is encoded by the Kcnma1 gene, and a splicing sequence defines its mitochondrial location. Proc. Natl. Acad. Sci. USA.

[20] Colbeau A., Nachbaur J., Vignais P. (1971). Enzymac characterization and lipid composition of rat liver subcellular membranes. Biochim. Biophys. Acta (BBA) Biomembr..

[21] Tillman T.S., Cascio M. (2003). Effects of membrane lipids on ion channel structure and function. Cell Biochem. Biophys..

[22] Duncan A.L., Reddy T., Koldsø H., Hélie J., Fowler P.W., Chavent M., Sansom M.S. (2017). Protein crowding and lipid complexity influence the nanoscale dynamic organization of ion channels in cell membranes. Sci. Rep..

[23] Cordero-Morales J.F., Vásquez V. (2018). How lipids contribute to ion channel function, a fat perspective on direct and indirect interactions. Curr. Opin. Struct. Biol..

[24] Lee A.G. (2004). How lipids affect the activities of integral membrane proteins. Biochim. Biophys. Acta (BBA) Biomembr..

[25] Brown M.F., Chawla U., Perera S.M. (2017). Membrane Lipid-Protein Interactions. The Biophysics of Cell Membranes.

[26] Laskowski M., Augustynek B., Bednarczyk P., Żochowska M., Kalisz J., O’rourke B., Szewczyk A., Kulawiak B. (2019). Single-channel properties of the ROMK-pore-forming subunit of the mitochondrial ATP-sensitive potassium channel. Int. J. Mol. Sci..

[27] Bednarczyk P., Wieckowski M.R., Broszkiewicz M., Skowronek K., Siemen D., Szewczyk A. (2013). Putative structural and functional coupling of the mitochondrial BK Ca channel to the respiratory chain. PLoS ONE.

[28] Rosa P., Sforna L., Carlomagno S., Mangino G., Miscusi M., Pessia M., Franciolini F., Calogero A., Catacuzzeno L. (2017). Overexpression of large-conductance calcium-activated potassium channels in human glioblastoma stem-like cells and their role in cell migration. J. Cell. Physiol..

[29] Ransom C.B., Liu X., Sontheimer H. (2002). BK channels in human glioma cells have enhanced calcium sensitivity. Glia.

[30] Molenaar R.J. (2011). Ion channels in glioblastoma. ISRN Neurol..

[31] Weaver A.K., Bomben V.C., Sontheimer H. (2006). Expression and function of calcium-activated potassium channels in human glioma cells. Glia.

[32] Edalat L., Stegen B., Klumpp L., Haehl E., Schilbach K., Lukowski R., Kühnle M., Bernhardt G., Buschauer A., Zips D. (2016). BK K+ channel blockade inhibits radiation-induced migration/brain infiltration of glioblastoma cells. Oncotarget.

[33] Rosa P., Catacuzzeno L., Sforna L., Mangino G., Carlomagno S., Mincione G., Petrozza V., Ragona G., Franciolini F., Calogero A. (2018). BK channels blockage inhibits hypoxia-induced migration and chemoresistance to cisplatin in human glioblastoma cells. J. Cell. Physiol..

[34] Kicinska A., Kampa R.P., Daniluk J., Sek A., Jarmuszkiewicz W., Szewczyk A., Bednarczyk P. (2020). Regulation of the mitochondrial BKCa channel by the citrus flavonoid naringenin as a potential means of preventing cell damage. Molecules.

[35] Frankenreiter S., Bednarczyk P., Kniess A., Bork N.I., Straubinger J., Koprowski P., Wrzosek A., Mohr E., Logan A., Murphy M.P. (2017). cGMP-elevating compounds and ischemic conditioning provide cardioprotection against ischemia and reperfusion injury via cardiomyocyte-specific BK channels. Circulation.

[36] Laskowski M., Augustynek B., Kulawiak B., Koprowski P., Bednarczyk P., Jarmuszkiewicz W., Szewczyk A. (2016). What do we not know about mitochondrial potassium channels?. Biochim. Biophys. Acta (BBA) Bioenerg..

[37] Aldape K., Brindle K.M., Chesler L., Chopra R., Gajjar A., Gilbert M.R., Gottardo N., Gutmann D.H., Hargrave D., Holland E.C. (2019). Challenges to curing primary brain tumours. Nat. Rev. Clin. Oncol..

[38] Chinot O.L., Wick W., Mason W., Henriksson R., Saran F., Nishikawa R., Carpentier A.F., Hoang-Xuan K., Kavan P., Cernea D. (2014). Bevacizumab plus radiotherapy–temozolomide for newly diagnosed glioblastoma. N. Engl. J. Med..

[39] Gilbert M.R., Dignam J.J., Armstrong T.S., Wefel J.S., Blumenthal D.T., Vogelbaum M.A., Colman H., Chakravarti A., Pugh S., Won M. (2014). A randomized trial of bevacizumab for newly diagnosed glioblastoma. N. Engl. J. Med..

[40] Wang H., Xu T., Huang Q., Jin W., Chen J. (2020). Immunotherapy for malignant glioma: Current status and future directions. Trends Pharmacol. Sci..

[41] Szewczyk A., Skalska J., Głąb M., Kulawiak B., Malińska D., Koszela-Piotrowska I., Kunz W.S. (2006). Mitochondrial potassium channels: From pharmacology to function. Biochim. Biophys. Acta (BBA) Bioenerg..

[42] Szewczyk A., Kajma A., Malinska D., Wrzosek A., Bednarczyk P., Zabłocka B., Dołowy K. (2010). Pharmacology of mitochondrial potassium channels: Dark side of the field. FEBS Lett..

[43] Singh H., Stefani E., Toro L. (2012). Intracellular BKCa (iBKCa) channels. J. Physiol..

[44] Walewska A., Kulawiak B., Szewczyk A., Koprowski P. (2018). Mechanosensitivity of mitochondrial large-conductance calcium-activated potassium channels. Biochim. Biophys. Acta (BBA) Bioenerg..

[45] Ransom C.B., Sontheimer H. (2001). BK channels in human glioma cells. J. Neurophysiol..

[46] Hurst H.E. (1956). The problem of long-term storage in reservoirs. Hydrol. Sci. J..

[47] Peng C.K., Buldyrev S.V., Havlin S., Simons M., Stanley H.E., Goldberger A.L. (1994). Mosaic organization of DNA nucleotides. Phys. Rev..

[48] Kantelhardt J.W., Zschiegner S.A., Koscielny-Bunde E., Havlin S., Bunde A., Stanley H.E. (2002). Multifractal detrended fluctuation analysis of nonstationary time series. Phys. A Stat. Mech. Appl..

[49] Nogueira R., Varanda W., Liebovitch L. (1995). Hurst analysis in the study of ion channel kinetics. Braz. J. Med. Biol. Res. Rev. Bras. Pesqui. Med. Biol..

[50] De Oliveira R.C., Barbosa C., Consoni L., Rodrigues A., Varanda W., Nogueira R. (2006). Long-term correlation in single calcium-activated potassium channel kinetics. Phys. A Stat. Mech. Appl..

[51] Varanda W.A., Liebovitch L.S., Figueiroa J.N., Nogueira R.A. (2000). Hurst analysis applied to the study of single calcium-activated potassium channel kinetics. J. Theor. Biol..

[52] Borys P. (2020). Long term Hurst memory that does not die at long observation times—Deterministic map to describe ion channel activity. Chaos Solitons Fractals.

[53] Bahramian A., Nouri A., Baghdadi G., Gharibzadeh S., Towhidkhah F., Jafari S. (2019). Introducing a chaotic map with a wide range of long-term memory as a model of patch-clamped ion channels current time series. Chaos Solitons Fractals.

[54] Wawrzkiewicz A., Pawelek K., Borys P., Dworakowska B., Grzywna Z.J. (2012). On the simple random-walk models of ion-channel gate dynamics reflecting long-term memory. Eur. Biophys. J..

[55] Siwy Z., Mercik S., Weron K., Ausloos M. (2001). Application of dwell-time series in studies of long-range correlation in single channel ion transport: Analysis of ion current through a big conductance locust potassium channel. Phys. A Stat. Mech. Appl..

[56] Siwy Z., Ausloos M., Ivanova K. (2002). Correlation studies of open and closed state fluctuations in an ion channel: Analysis of ion current through a large-conductance locust potassium channel. Phys. Rev. E.

[57] Lan T.H., Gao Z.Y., Abdalla A.N., Cheng B., Wang S. (2008). Detrended fluctuation analysis as a statistical method to study ion single channel signal. Cell Biol. Int..

[58] Kazachenko V., Astashev M., Grinevich A. (2007). Multifractal analysis of *K*^+^ channel activity. Biochem. (Mosc.) Suppl. Ser. A Membr. Cell Biol..

[59] Wawrzkiewicz-Jałowiecka A., Trybek P., Dworakowska B., Machura Ł. (2020). Multifractal Properties of BK Channel Currents in Human Glioblastoma Cells. J. Phys. Chem. B.

[60] Mercik S., Weron K., Siwy Z. (1999). Statistical analysis of ionic current fluctuations in membrane channels. Phys. Rev. E.

[61] Mukli P., Nagy Z., Eke A. (2015). Multifractal formalism by enforcing the universal behavior of scaling functions. Phys. A Stat. Mech. Its Appl..

[62] McKinney W. Data structures for statistical computing in python. Proceedings of the 9th Python in Science Conference.

[63] Colquhoun D., Hawkes A. (1987). A note on correlations in single ion channel records. Proc. R. Soc. Lond. Ser. B Biol. Sci..

[64] Colquhoun D., Hawkes A.G. (1995). The principles of the stochastic interpretation of ion-channel mechanisms. Single-Channel Recording.

[65] Geng Y., Magleby K.L. (2015). Single-channel kinetics of BK (Slo1) channels. Front. Physiol..

[66] Rothberg B.S., Magleby K.L. (1999). Gating Kinetics of Single Large-Conductance Ca^2+^-Activated *K*^+^ Channels in High Ca^2+^ Suggest a Two-Tiered Allosteric Gating Mechanism. J. Gen. Physiol..

[67] Cui J., Aldrich R.W. (2000). Allosteric linkage between voltage and Ca^2+^-dependent activation of BK-type mslo1 *K*^+^ channels. Biochemistry.

[68] Bunde A., Havlin S. (2012). Fractals and Disordered Systems.

[69] Chen Y., Huang L. (2018). Spatial measures of urban systems: From entropy to fractal dimension. Entropy.

[70] Peyronnet R., Nerbonne J.M., Kohl P. (2016). Cardiac mechano-gated ion channels and arrhythmias. Circ. Res..

[71] N’gouemo P. (2011). Targeting BK (big potassium) channels in epilepsy. Expert Opin. Ther. Targets.

